# Poretools: a toolkit for analyzing nanopore sequence data

**DOI:** 10.1093/bioinformatics/btu555

**Published:** 2014-08-20

**Authors:** Nicholas J. Loman, Aaron R. Quinlan

**Affiliations:** ^1^Institute of Microbiology and Infection, University of Birmingham, Birmingham B15 2TT, UK and ^2^Department of Public Health Sciences, University of Virginia, Charlottesville 22932, VA, USA

## Abstract

**Motivation:** Nanopore sequencing may be the next disruptive technology in genomics, owing to its ability to detect single DNA molecules without prior amplification, lack of reliance on expensive optical components, and the ability to sequence long fragments. The MinION™ from Oxford Nanopore Technologies (ONT) is the first nanopore sequencer to be commercialized and is now available to early-access users. The MinION™ is a USB-connected, portable nanopore sequencer that permits real-time analysis of streaming event data. Currently, the research community lacks a standardized toolkit for the analysis of nanopore datasets.

**Results:** We introduce poretools, a flexible toolkit for exploring datasets generated by nanopore sequencing devices from MinION™ for the purposes of quality control and downstream analysis. Poretools operates directly on the native FAST5 (an application of the HDF5 standard) file format produced by ONT and provides a wealth of format conversion utilities and data exploration and visualization tools.

**Availability and implementation:** Poretools is an open-source software and is written in Python as both a suite of command line utilities and a Python application programming interface. Source code is freely available in Github at https://www.github.com/arq5x/poretools

**Contact:** n.j.loman@bham.ac.uk and aaronquinlan@gmail.com

**Supplementary information:** An IPython notebook demonstrating the functionality of poretools is in Github. Complete documentation is available at http://poretools.readthedocs.org.

## 1 INTRODUCTION

DNA sequencing with biological nanopores was proposed almost 20 years ago (Church *et al.*, 1995). This approach relies on the direct electrical detection of single DNA strands in contact with an individual pore. Single molecule detection and the absence of a prior amplification step means that extremely long fragments can be sequenced without any loss in quality. In May 2014, Oxford Nanopore Technologies released MinION™, the first commercially available nanopore DNA sequencing device. MinION™ is noteworthy for its portability, size (around the same length as an iPhone™) and USB 3.0 connectivity, meaning it can be run on a standard Internet-connected laptop. Research groups throughout the world are actively evaluating this device for a broad range of applications. Sequencing with the MinION yields raw signals reflecting modulation of the ionic current at each pore by a DNA molecule. The resulting time-series of nanopore translocation, ‘events’, are base-called by proprietary software running as a cloud service. The resulting files for each sequenced read are stored in ‘FAST5’ format, an application of the HDF5 format. However, at present, no specific software is available to facilitate downstream analyses starting with this file format.

## 2 FEATURES AND METHODS

We have developed poretools, an open-source software toolkit that addresses the pressing need for methods to manipulate the FAST5 format and permit explorations of the raw nanopore event data and the resulting DNA sequences. Poretools provides an extensive set of data analysis methods that operate directly on either a single FAST5 file or a set of files from one or more sequencing runs. A Python programming library is provided to facilitate access to the FAST5 file structure and enable other researchers to extend the tools and create new analytical methods. In the following sections, we summarize the functionality currently available in poretools.

### 2.1 Format conversion

The most fundamental functionality provided by poretools is the ability to convert the output data resulting from a MinION run from HDF5/FAST5 format to either FASTA or FASTQ format to facilitate analyses with sequence alignment and/or assembly software. This is accomplished with the fasta and fastq commands in the poretools suite.

poretools **fasta** /path/to/fast5/example.fast5
poretools **fastq** /path/to/fast5/example.fast5

At the time of writing, each MinION run generates individual HDF5/FAST5 files for each sequenced read. Consequently, there are often tens of thousands of individual files that must be stored for a single experiment. Poretools provides two different strategies for facilitating the analysis of such datasets. The first approach allows one to execute a poretools command on an entire directory of FAST5 files.

poretools **fastq** /path/to/fast5/directory/

Alternatively, we provide a utility to combine a set of HDF5/FAST5 files into a single TAR file. This allows an entire run to be archived into one file and once combined, all other poretools commands are able to operate on each HDF5/FAST5 file therein. For example:

poretools combine -o run.tar /path/to/fast5/directory/poretools **fastq** run.tar

### 2.2 Data exploration and visualization

**Fig. 1.**
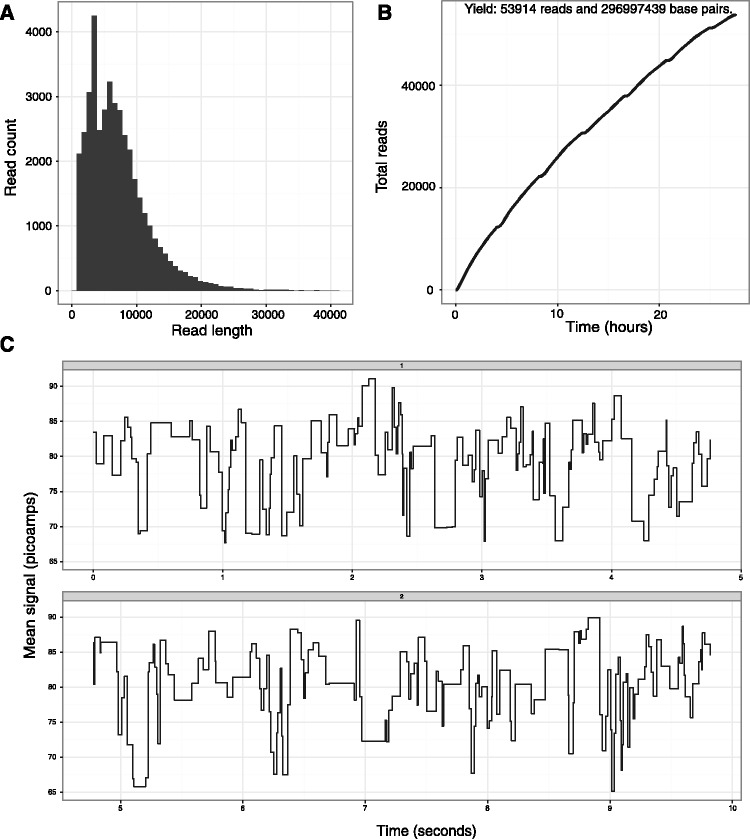
Example poretools visualizations from a set of FAST5 files generated by a single MinION™ run. Panel **A** shows a histogram of read lengths. Panel **B** shows a collector’s curve of reads over time. Panel **C** shows an example squiggle plot of detected event transitions originating from MinION™

There is a need to visualize MinION™ run performance to assess its quality and troubleshoot different fragmentation and library preparation strategies. Poretools provides two utilties, hist and yield_plot, that characterize the fragment size distribution and display a collector’s curve of the overall sequencing yield, respectively. Example commands are provided here, with corresponding figures shown (Fig. 1A and B).

poretools **hist** /path/to/fast5/directory/
poretools **yield_plot** /path/to/fast5/directory/

As summarized in Table 1, poretools also provides several utilities for extracting the low-level details that led to each base called sequence (see Table 1 for details). In particular, the events utility reports the mean current observed for each nanopore translocation event, as well as the time (in milliseconds) of each event and the k-mer that was predicted to have occupied the nanopore during the event. The squiggle utility permits visualization of this information (Fig. 1C). The Oxford Nanopore base-calling software uses a Hidden Markov Model to predict a fragment’s sequence based on this event data. We anticipate that the events utility (and others) will help developers explore improved base-calling strategies.

**Table 1.** Summary of currently supported operations in poretools

Command	Description
combine	Combine a set of FAST5 files in a TAR archive.
events	Extract each nanopore event for each read.
fasta	Extract FASTA sequences from a set of FAST5 files.
fastq	Extract FASTQ sequences from a set of FAST5 files.
hist	Plot read size histogram for a set of FAST5 files.
nucdist	Measure the nucleotide composition.
qualdist	Measure the quality score composition.
readstats	Extract signal information for each read over time.
squiggle	Plot the observed signals for FAST5 reads.
stats	Get read size stats for a set of FAST5 files.
tabular	Extract sequence information in TAB delimited format
times	Return the start times from a set of FAST5 files.
winner	Extract the longest read from a set of FAST5 files.
yield_plot	Plot the sequencing yield over time.

### 2.3 Python library for data analysis

The utilities provided in the poretools suite will inevitably prove to be insufficient for every analysis that a researcher wishes to conduct. Recognizing this, we have developed a Python programming interface that researchers can use to directly access the sequence data, the raw nanopore event data and other metadata (e.g. the flowcell and run identifiers) contained in one or more FAST5 files. To demonstrate of the Python interface, the following code reports the start time, the specific nanopore and the based-called sequence for each FAST5 file in a sequencing run.

**from** poretools **import** Fast5FileSet
fast5s = Fast5FileSet(’/path/to/fast5/files/’)
**for** fast5 **in** fast5s:
 start = fast5.get_start_time()
 porenum = fast5.get_channel_number()
 fq = fast5.get_fastq()
 **print** porenum, start, fq.seq, fq.qual
 fast5.close()

## 3 DISCUSSION

The poretools software helps solve pressing requirements for analysis of nanopore sequencing data. By focusing on the Python development environment and adopting expected interface conventions as popularized by other popular bioinformatics tools such as samtools (Li *et al.*, 2010) and bedtools (Quinlan *et al.*, 2009), we expect that users will be able to rapidly exploit the functionality offered by this software. We anticipate that other toolkits will become available written in other programming languages. Further efforts are required for downstream analysis for common tasks including alignment and *de novo* assembly of both event and base-called sequence data from this platform.
